# *In Silico* Investigation of Potential mTOR Inhibitors from Traditional Chinese Medicine for Treatment of Leigh Syndrome

**DOI:** 10.1155/2014/139492

**Published:** 2014-06-23

**Authors:** Kuan-Chung Chen, Wen-Yuan Lee, Hsin-Yi Chen, Calvin Yu-Chian Chen

**Affiliations:** ^1^School of Pharmacy, China Medical University, Taichung 40402, Taiwan; ^2^School of Medicine, College of Medicine, China Medical University, Taichung 40402, Taiwan; ^3^Department of Biomedical Informatics, Asia University, Taichung 41354, Taiwan; ^4^Department of Neurosurgery, China Medical University Hospital, Taichung 40447, Taiwan; ^5^Human Genetic Center, Department of Medical Research, China Medical University Hospital, Taichung, Taiwan; ^6^Research Center for Chinese Medicine & Acupuncture, China Medical University, Taichung 40402, Taiwan

## Abstract

A recent research demonstrates that the inhibition of mammalian target of rapamycin (mTOR) improves survival and health for patients with Leigh syndrome. mTOR proteins can be treated as drug target proteins against Leigh syndrome and other mitochondrial disorders. In this study, we aim to identify potent TCM compounds from the TCM Database@Taiwan as lead compounds of mTOR inhibitors. PONDR-Fit protocol was employed to predict the disordered disposition in mTOR protein before virtual screening. After virtual screening, the MD simulation was employed to validate the stability of interactions between each ligand and mTOR protein in the docking poses from docking simulation. The top TCM compounds, picrasidine M and acerosin, have higher binding affinities with target protein in docking simulation than control. There have H-bonds with residues Val2240 and *π* interactions with common residue Trp2239. After MD simulation, the top TCM compounds maintain similar docking poses under dynamic conditions. The top two TCM compounds, picrasidine M and acerosin, were extracted from *Picrasma quassioides* (D. Don) Benn. and *Vitex negundo* L. Hence, we propose the TCM compounds, picrasidine M and acerosin, as potential candidates as lead compounds for further study in drug development process with the mTOR protein against Leigh syndrome and other mitochondrial disorders.

## 1. Introduction

Leigh syndrome is a rare fatal prototypical mitochondrial disorder for children [1, 2]. It is a serious disorder for children as it can lead to death within the first few years of life [3, 4]. Recently, increasing numbers of pathogeneses for diseases have been identified [5, 6] to identify the potential target proteins for drug design [7–10]. A recent research demonstrates that the inhibition of mammalian target of rapamycin (mTOR) improves survival and health for patients with Leigh syndrome [11]. The mTOR proteins can be treated as drug target proteins against Leigh syndrome and other mitochondrial disorders [12–14].

Nowadays, compounds extracted from traditional Chinese medicine (TCM) have shown their potential to be lead compounds against cancers [15–17], diabetes [18], inflammation [19], metabolic syndrome [20], stroke [21, 22], viral infection [23, 24], and many different diseases [25, 26]. In this study, we aim to identify potent TCM compounds from the TCM Database@Taiwan [27] as lead compounds of mTOR inhibitors, in order to improve the development of TCM compounds. As structural disordered disposition in the protein may be the cause of side effect and decrease of occupancy for ligand to bind with target protein [28], PONDR-Fit protocol was employed to predict the disordered disposition in mTOR protein before virtual screening. After virtual screening, the MD simulation was employed to validate the stability of interactions between each ligand and mTOR protein in the docking poses from docking simulation.

## 2. Materials and Methods

### 2.1. Data Collection

The X-ray crystallography structure of the mammalian target of rapamycin (mTOR) was obtained from RCSB Protein Data Bank with PDB ID 4JSX [29]. To predict the disordered residues in mTOR protein, PONDR-Fit [30] protocol was employed with the sequence from Swiss-Prot (UniProtKB: P42345). The X-ray crystallography structure of mTOR protein was prepared by Prepare Protein Module in Discovery Studio 2.5 (DS2.5) to remove crystal water and protonate the final structure with Chemistry at HARvard Macromolecular Mechanics (CHARMM) force field [31]. The TCM compounds from TCM Database@Taiwan [27] were prepared by Prepare Ligand Module in DS2.5 to protonate their final structures and filter by Lipinski's rule of five [32]. The binding site for virtual screening was defined by the volume of the cocrystallized mTOR inhibitor, Torin2.

### 2.2. Docking Simulation

LigandFit protocol [33] in DS 2.5 was employed to redock cocrystallized mTOR inhibitor, Torin2, and dock the TCM compounds into the binding site defined above. The LigandFit protocol was performed using a shape filter and Monte-Carlo ligand conformation generation and then optionally minimized the docking poses with CHARMM force field [31]. Similar poses were filtered by the clustering algorithm. Each docking pose was evaluated by three scoring functions, -PLP1, -PLP2, and Dock Score.

### 2.3. Molecular Dynamics (MD) Simulation

Gromacs 4.5.5 [34] is a program used to perform the molecular dynamics (MD) simulation using classical molecular dynamics theory. In preparation section, the pdb2gmx protocol of Gromacs and the SwissParam program [35] were performed to provide topology and parameters of mTOR proteins with CHARMM27 force field and each ligand with CHARMM, respectively. For solvation, a cubic box was defined based upon the edge approximately 12 Å from the protein complexes periphery and solvated with TIP3P water model and 0.145 M NaCl model. For minimization, a maximum of 5,000 steps using steepest descents [36] minimization was employed to remove bad van der Waals contacts. Gromacs program utilizing position-restrained molecular dynamics with the Linear Constraint algorithm for the equilibration was performed with NVT equilibration, Berendsen weak thermal coupling method, and particle mesh Ewald method. For production, a total of 5000 ps production simulation with time step in unit of 2 fs was performed with NPT ensembles and particle mesh Ewald (PME) option. A series of protocols in Gromacs program was employed to analyze the MD trajectories.

## 3. Results and Discussion

### 3.1. Disordered Protein Prediction

**Figure 1:**
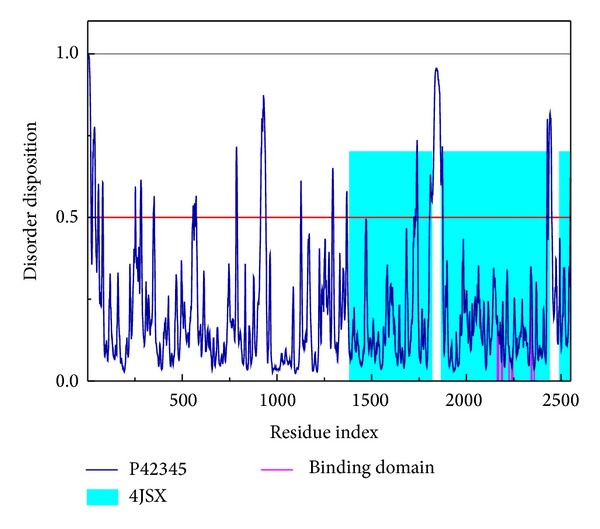
Disordered disposition predicted by PONDR-Fit.

The disordered disposition of residues in mTOR protein was predicted by PONDR-Fit protocol with the sequence of mTOR protein from Swiss-Prot (UniProtKB: P42345). As illustrated in Figure 1, the key residues in the binding site of mTOR protein were not laid in the disordered area (>0.5). It indicates that the mTOR protein expresses a stable binding domain in protein folding and is suitable for docking simulation.

### 3.2. Docking Simulation

**Figure 2:**
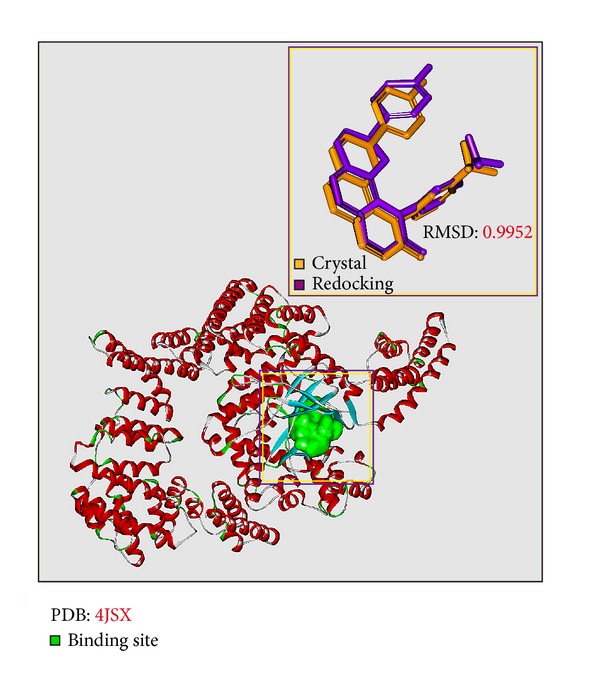
Binding site of mTOR protein defined as the volume of Torin2 and root-mean-square deviation value between crystallized structure (orange) and docking pose (violet) of Torin2.

**Figure 3:**
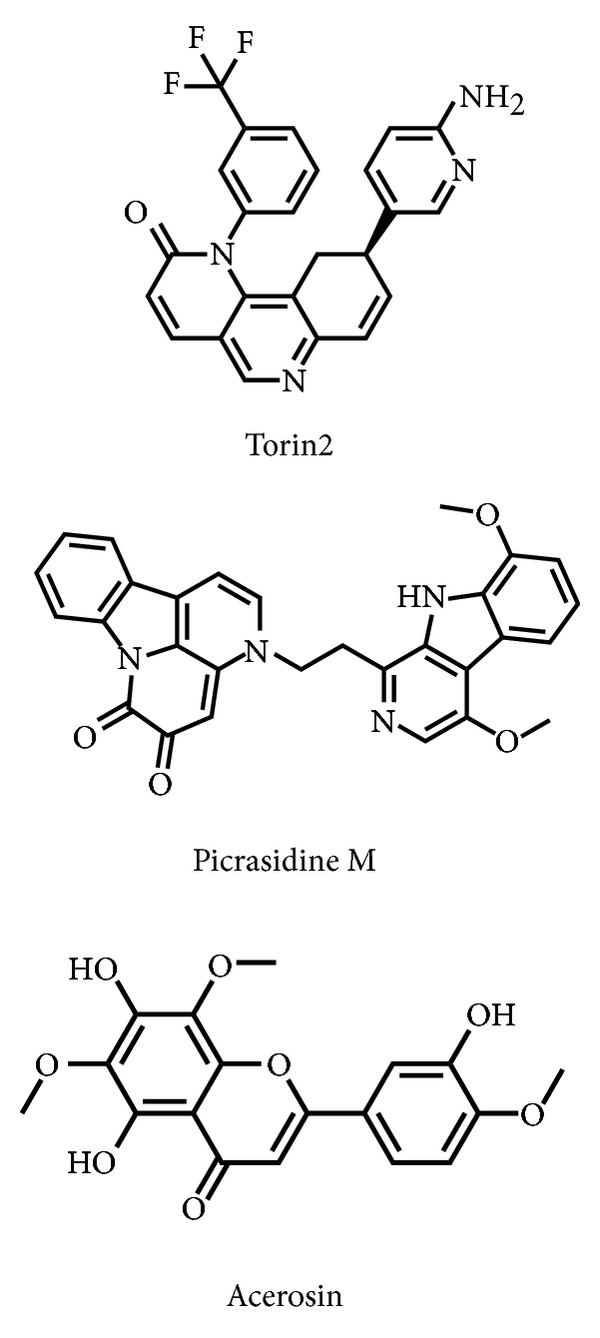
Chemical scaffold of controls and top two TCM candidates.

**Figure 4:**
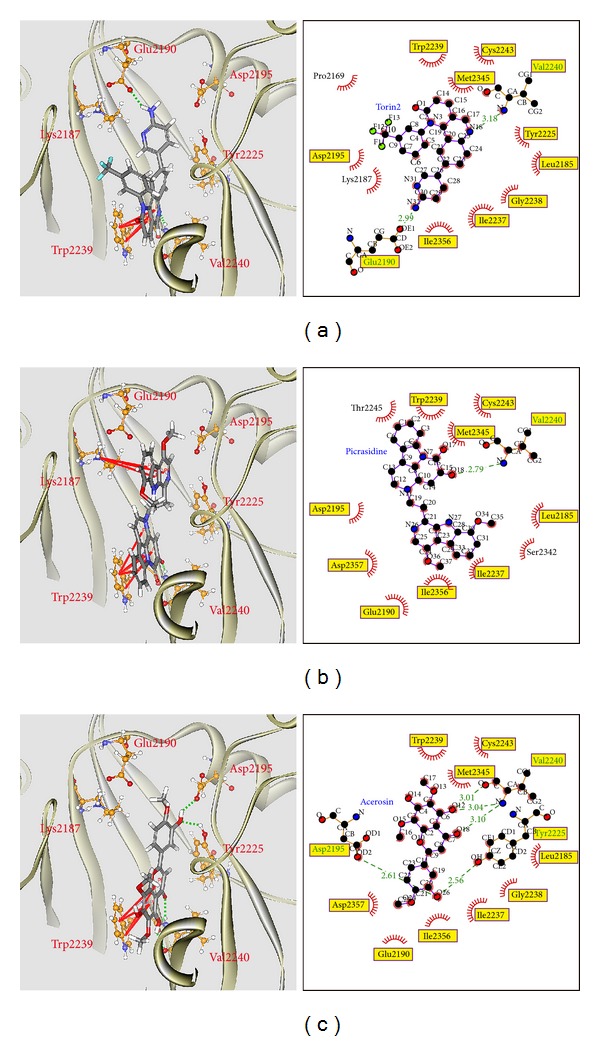
Docking pose of mTOR protein complexes with (a) Torin2, (b) picrasidine M, and (c) acerosin.

To validate the accuracy of LigandFit protocol, the cocrystallized mTOR inhibitor, Torin2, was redocked into the binding site of mTOR protein. As root-mean-square deviation (RMSD) value between crystallized structure and docking pose of Torin2 is 0.5834 (Figure 2), LigandFit protocol is suitable for virtual screening with mTOR protein. After virtual screening, the chemical scaffold top TCM compounds ranked by Dock Score [33] and control, Torin2, are shown in Table 1. The top two TCM compounds, picrasidine M and acerosin, were extracted from* Picrasma quassioides *(D. Don) Benn. and* Vitex negundo *L. The chemical scaffold top TCM compounds and control are illustrated in Figure 3. According to the docking poses in Figure 4, the candidate compounds and Torin2 have hydrogen bonds (H-bonds) and *π* interactions with common residues Val2240 and Trp2239, respectively. In addition, they have hydrophobic contacts with residues Glu2190, Asp2195, Leu2185, Tyr2225, Ile2237, Gly2238, Trp2239, Val2240, Cys2243, Met2345, and Ile2356.

### 3.3. Molecular Dynamics Simulation

**Figure 5:**
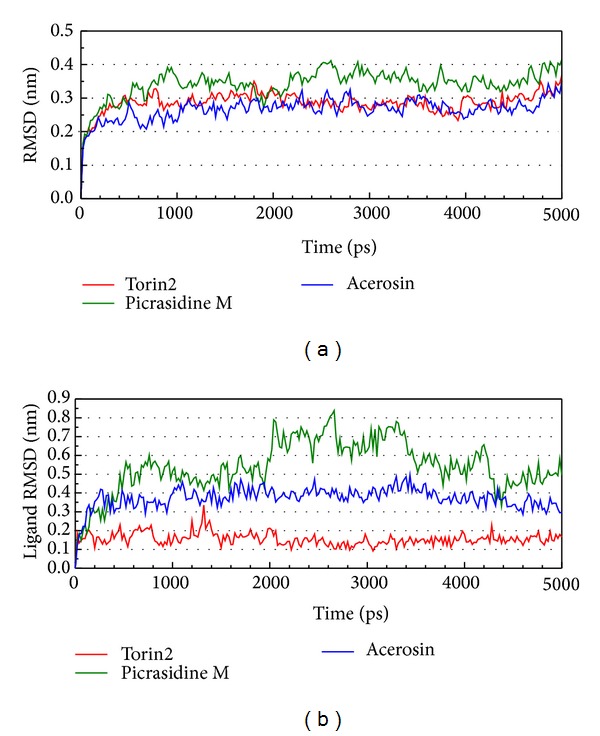
Root-mean-square deviations in units of nm for protein and ligand over 5000 ps of MD simulation for mTOR protein complexes with Torin2, picrasidine M, and acerosin.

**Figure 6:**
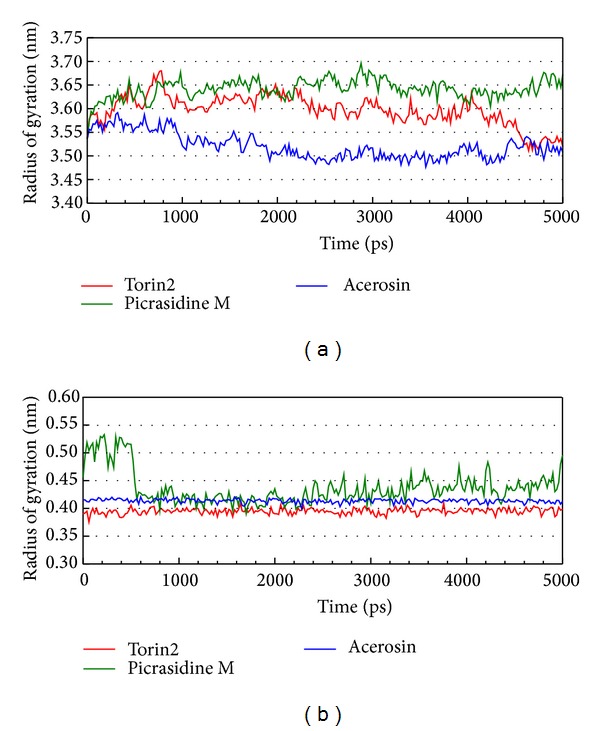
Variation of radii of gyration for (a) protein and (b) ligands for mTOR protein complexes with Torin2, picrasidine M, and acerosin over 5000 ps of MD simulation.

**Figure 7:**
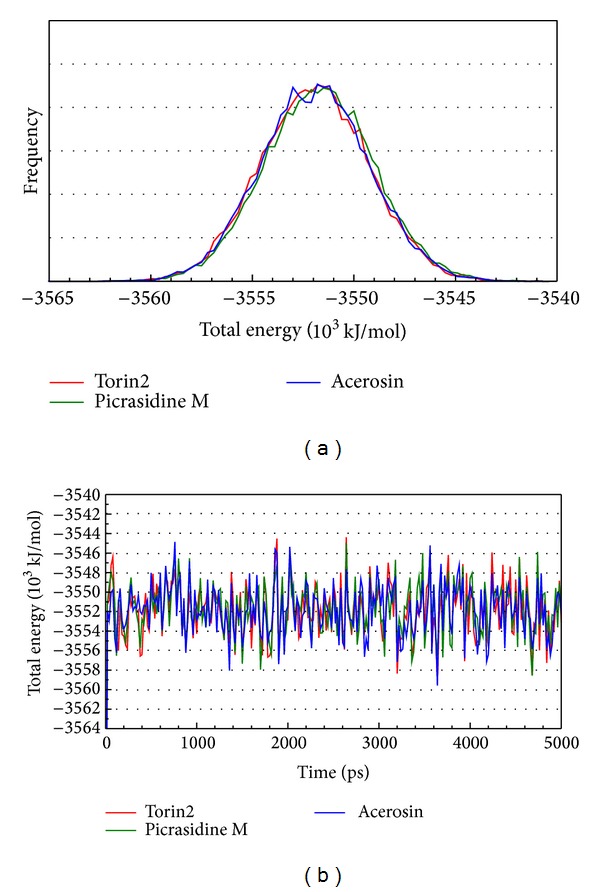
(a) Distribution and (b) variation of total energy for mTOR protein complexes with Torin2, picrasidine M, and acerosin over 5000 ps of MD simulation.

**Figure 8:**
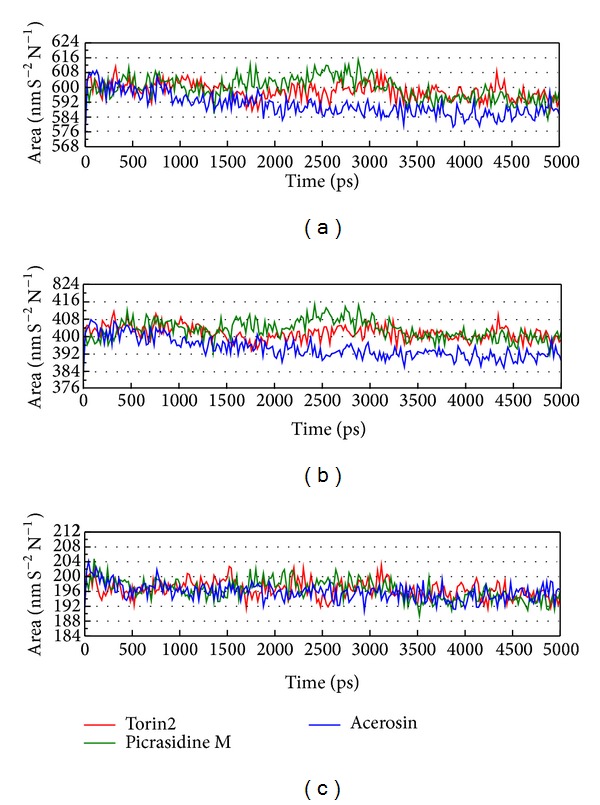
Variation of (a) total solvent accessible surface area, (b) hydrophobic surface area, and (c) hydrophilic surface area for mTOR protein complexes with Torin2, picrasidine M, and acerosin over 5000 ps of MD simulation.

LigandFit protocol performed a docking simulation with a rigid body of mTOR proteins, so the conformation of the mTOR protein may modify under dynamic conditions. For this reason, MD simulation was performed to validate the stability of interactions between mTOR proteins and each ligand. The atomic fluctuations of mTOR proteins and ligands in protein complexes with picrasidine M, acerosin, and control were displayed in Figure 5. It shows that mTOR proteins tend to be stable after MD simulation. They indicate that the atoms of picrasidine M have a sharp fluctuation before the system tends to be stable. The variation of radii of gyration for protein and ligand over 5000 ps MD simulation was displayed in Figure 6. They show that the radii of gyration for mTOR protein complexes with ligand except picrasidine M have decreased after MD simulation. For the total energy of each protein complex, there is no significant variation during MD simulation (Figure 7). The variation of solvent accessible surface area over 5000 ps MD simulation shown in Figure 8 indicates that docking with picrasidine M, acerosin, and Torin2 would not affect the solvent accessible surface of mTOR protein under dynamic conditions.

**Figure 9:**
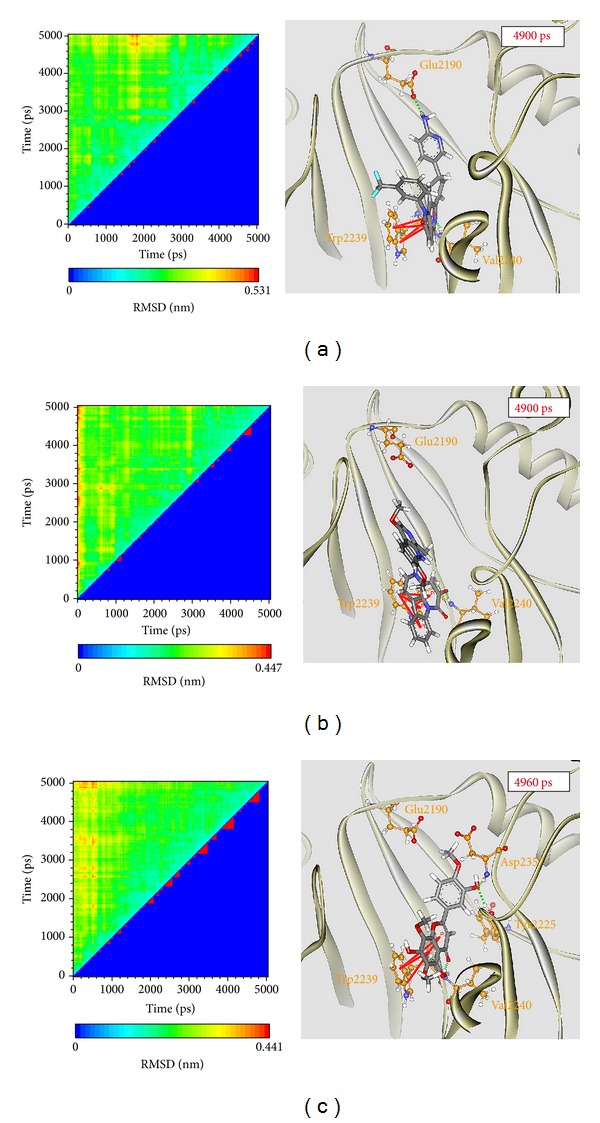
Left: root-mean-square deviation value (upper left half) and graphical depiction of the clusters with cutoff 0.12 nm (lower right half) and right: docking poses of middle RMSD structure in the major cluster for mTOR protein complexes with (a) Torin2, (b) picrasidine M, and (c) acerosin.

To compare the variation of docking poses in docking simulation and after MD simulation, we identify the representative structures of mTOR protein complexes using the RMSD values and graphical depiction of the clusters analysis with a RMSD cutoff of 0.12 nm and illustrated the docking poses of the representative structures in Figure 9. The H-bond occupancy for key residues of mTOR protein with each ligand is listed in Table 2. For Torin2, it has stable H-bonds with residues Glu2190 and Val2240 and *π* interactions with residue Trp2239. For the TCM candidates, they also have stable H-bonds with residues Val2240 and *π* interactions with residue Trp2239 as Torin2, which indicate that they have similar docking pose and effects in mTOR protein.

## 4. Conclusion

This study aims to investigate the potent lead TCM candidates for mTOR protein inhibitors against Leigh syndrome and other mitochondrial disorders. The top TCM compounds, picrasidine M and acerosin, have higher binding affinities with target protein in docking simulation than control. There have H-bonds with residues Val2240, *π* interactions with common residue Trp2239, and hydrophobic contacts with residues Glu2190, Asp2195, Leu2185, Tyr2225, Ile2237, Gly2238, Trp2239, Val2240, Cys2243, Met2345, and Ile2356. After MD simulation, the top TCM compounds maintain similar docking poses under dynamic conditions as control. In addition, the top two TCM compounds, picrasidine M and acerosin, were extracted from* Picrasma quassioides *(D. Don) Benn. and* Vitex negundo *L. Hence, we propose the TCM compounds, picrasidine M and acerosin, as potential candidates as lead compounds for further study in drug development process with the mTOR protein against Leigh syndrome and other mitochondrial disorders.

## Figures and Tables

**Table 1:** Scoring functions of top candidates and Torin2 from TCM database screening.

Name	Resource	-PLP1	-PLP2	Dock Score
Picrasidine M	*Picrasma quassioides* (D. Don) Benn.	118.22	104.11	88.913
Acerosin	*Vitex negundo* L.	104.12	108.76	78.088
Psychotrine	*Alangium lamarckii *	107.12	97.2	75.322
3,5,6-Trihydroxy-3^'^,4^'^,7-trimethoxyflavone	*Citrus medica* L. var.* sarcodactylis *(Noot.) Swingie	106.07	105.45	73.906
Torin2*		**115.23**	**107.26**	**55.507**

*Control.

**Table 2:** H-bond occupancy for key residues of mTOR protein with Torin2 and top TCM compounds over 5000 ps of molecular dynamics simulation.

Name	H-bond interaction		Occupancy
Torin2	Lys2187:HZ3	/N31	2%
	Lys2187:HZ3	/N32	35%
	Glu2190:OE1	/H48	35%
	Glu2190:OE2	/H48	4%
	Val2240:HN	/N18	100%
	Thr2245:HG1	/O1	7%
	Asp2357:OD1	/H48	27%
	Asp2357:OD2	/H48	4%

Picrasidine M	Ser2165:HG1	/O36	2%
	Lys2187:HZ3	/O36	4%
	Trp2239:HE1	/O17	6%
	Val2240:HN	/O17	29%
	Val2240:HN	/O18	30%
	Thr2245:HG1	/O34	1%
	His2340:HE2	/O36	2%
	Ser2342:HG1	/O34	2%
	Asn2343:HD22	/N26	16%
	Asn2343:HD22	/O36	2%

Acerosin	Asp2195:OD2	/H42	38%
	Tyr2225:HH	/O26	96%
	Trp2239:HE1	/O13	4%
	Trp2239:NE1	/H28	3%
	Val2240:HN	/O12	10%
	Val2240:HN	/O18	96%
	Asp2357:HN	/O26	86%

H-bond occupancy cutoff: 0.3 nm.
